# Age-stratified Associations between Chronic Periodontal Disease and Clinical Outcomes in Hemodialysis Patients: Mortality, Pneumonia, and Fractures

**DOI:** 10.7150/ijms.116133

**Published:** 2025-10-24

**Authors:** Min-Tser Liao, Chien-Lin Lu, Ren-Yeong Huang, Joshua Wang, Cai-Mei Zheng, Yi-Chou Hou, Chen-Yen Tai, Kuo-Cheng Lu

**Affiliations:** 1 Department of Pediatrics, Taoyuan Armed Forces General Hospital, Taoyuan City 32551, Taiwan.; 2 Department of Pediatrics, Tri-Service General Hospital, National Defense Medical University, Taipei City 11490, Taiwan.; 3 School of Medicine, College of Medicine, Fu Jen Catholic University, New Taipei City 24205, Taiwan.; 4 Division of Nephrology, Department of Internal Medicine, Fu Jen Catholic University Hospital, Fu Jen Catholic University, New Taipei City 24352, Taiwan.; 5 Department of Periodontology, School of Dentistry, Tri-Service General Hospital, National Defense Medical University, Taipei City 11490, Taiwan.; 6 Department of Research, Taipei Tzu Chi Hospital, Buddhist Tzu Chi Medical Foundation, New Taipei City 23142, Taiwan.; 7 Division of Nephrology, Department of Internal Medicine, Shuang Ho Hospital, School of Medicine, College of Medicine, Taipei Medical University, New Taipei City 11031, Taiwan; TMU Research Centre of Urology and Kidney, Taipei Medical University, New Taipei City 11031, Taiwan.; 8 Division of Nephrology, Department of Internal Medicine, Cardinal-Tien Hospital, School of Medicine, College of Medicine, Fu Jen Catholic University, New Taipei City 24205, Taiwan.; 9 Division of Nephrology, Department of Medicine, Taipei Tzu Chi Hospital, Buddhist Tzu Chi Medical Foundation, New Taipei City 23142, Taiwan.

**Keywords:** Chronic periodontal disease, Hemodialysis, Mortality, Pneumonia, Bone Fractures, Systemic Inflammation

## Abstract

**Background:** Chronic periodontal disease (CPD) may contribute to systemic inflammation and adverse outcomes in patients undergoing maintenance hemodialysis, but age-specific risks remain unclear. We aimed to evaluate the association between CPD and clinical outcomes in hemodialysis patients, stratified by age.

**Methods:** We conducted a retrospective cohort study using the TriNetX Research Network. Adults aged 45-64 or ≥65 years initiating maintenance hemodialysis were included. CPD exposure was defined using ICD-10-CM codes documented within 6 months before or up to 5 years after hemodialysis initiation. Outcomes included all-cause mortality, pneumonia, fracture, and major adverse cardiovascular events (MACE) over a 5-year follow-up period. Propensity score matching was conducted within each age group using a comprehensive model that included age, sex, race, comorbidities, medications, and laboratory values. Adjusted hazard ratios (aHRs) with 95% confidence intervals (CIs) were estimated using Cox models. Subgroup and sensitivity analyses were conducted to assess robustness and effect modification.

**Results:** CPD was significantly associated with increased risks of all-cause mortality in both younger (aHR = 1.313, 95% CI: 1.129-1.527; p<0.001) and older patients (aHR = 1.126, 95% CI: 1.048-1.209; p=0.001), with stronger associations in the younger group. Subgroup analysis showed elevated mortality risks in younger males, females, and non-diabetic individuals. Pneumonia risk was significantly increased across both age groups, with particularly high risks in younger females and older males. CPD was also associated with a higher risk of fracture, particularly among older adults (aHR = 1.673, 95% CI: 1.375-2.036; p<0.001), with consistent findings across subgroups. No significant associations were observed between CPD and MACE in either age stratum. Sensitivity analyses adjusting for comorbidities and inflammatory markers supported the primary findings.

**Conclusion:** CPD is associated with increased risks of mortality, pneumonia, and fractures among hemodialysis patients, with variation by age and clinical subgroup. These findings support integrating periodontal screening and preventive strategies into hemodialysis care. Younger patients may benefit from early intervention to reduce infection and mortality risk, while older adults may require targeted skeletal protection.

## Introduction

Chronic kidney disease (CKD) and chronic periodontal disease (CPD) are prevalent conditions with significant public health implications. CKD, characterized by the gradual loss of kidney function, affects approximately 9.1% of the global population and is projected to become the fifth leading cause of death by 2040 1, 2. A major contributor to the increasing prevalence of CKD is population aging, as CKD incidence rises markedly with advancing age 3. Similarly, CPD, a chronic inflammatory condition affecting the supporting structures of teeth, affects over 50% of the global adult population and is increasingly associated with systemic conditions such as diabetes, cardiovascular disease, and CKD itself 4-6. The two diseases share common risk factors—including diabetes, hypertension, smoking, and chronic inflammation—and may interact through bidirectional mechanisms involving systemic inflammation, oxidative stress, and immune dysregulation 7.

In patients with end-stage renal disease (ESRD) receiving maintenance hemodialysis, systemic inflammation is a major driver of poor clinical outcomes, including increased risks of mortality and cardiovascular disease 8. These patients often present with multiple comorbidities such as malnutrition and impaired immune function, rendering them especially vulnerable to additional inflammatory stimuli. CPD, as a persistent source of systemic inflammation, may exacerbate these risks and contribute to adverse outcomes in this already high-risk population 9, 10.

Research examining the association between periodontitis and mortality in hemodialysis patients has yielded mixed results. Some studies suggest a significant association between periodontitis and increased mortality, highlighting oral health as a potentially modifiable risk factor 11. In contrast, the multinational ORAL Diseases in Hemodialysis (ORAL-D) study found no clear association after adjusting for confounders and even reported lower mortality among patients with periodontitis, possibly due to residual confounding, misclassification bias related to tooth loss, or limited follow-up duration 12. These discrepancies underscore the complexity of evaluating the impact of CPD in hemodialysis populations and emphasize the need for further well-designed investigations.

Importantly, most existing studies on periodontitis and mortality in hemodialysis patients have either focused predominantly on younger populations or failed to stratify adequately by age, leaving uncertainty regarding CPD's clinical impact on older adults 11-15. Elderly patients face unique challenges, including age-related immune dysregulation, increased competing mortality risks, and heightened vulnerability to skeletal fragility. These factors may amplify the detrimental effects of CPD, potentially leading to disproportionately higher risks of mortality, pneumonia, and fractures. Understanding these age-specific implications is essential for effective clinical management.

To address these gaps, the present study evaluates age-stratified associations between CPD and key clinical outcomes—including mortality, pneumonia, fractures, and major adverse cardiovascular events (MACE)—among hemodialysis patients aged 45-64 and ≥65 years. By clarifying how the systemic implications of CPD vary with age, this research aims to inform targeted oral health interventions and contribute to integrated care strategies that improve patient outcomes across age groups.

## Methods

### Study Design and Data Source

This retrospective cohort study utilized data from TriNetX, a federated global health research network providing access to deidentified electronic health records (EHRs) from a diverse group of healthcare organizations. The study specifically employed data from the Global Collaborative Network within TriNetX, comprising 143 healthcare organizations worldwide. TriNetX captures extensive structured patient data, including demographics, diagnoses, procedures, medications, and laboratory values.

The analysis was conducted on January 4, 2025, using the Compare Outcomes module within the TriNetX platform, which complies with the Health Insurance Portability and Accountability Act (HIPAA) and the General Data Protection Regulation (GDPR). The study adheres to the Strengthening the Reporting of Observational Studies in Epidemiology (STROBE) guidelines for observational research. Ethical approval was granted by the Taipei Tzu Chi Hospital Institutional Review Board (Approval Number: 14-IRB004).

### Study Population

**Figure 1:**
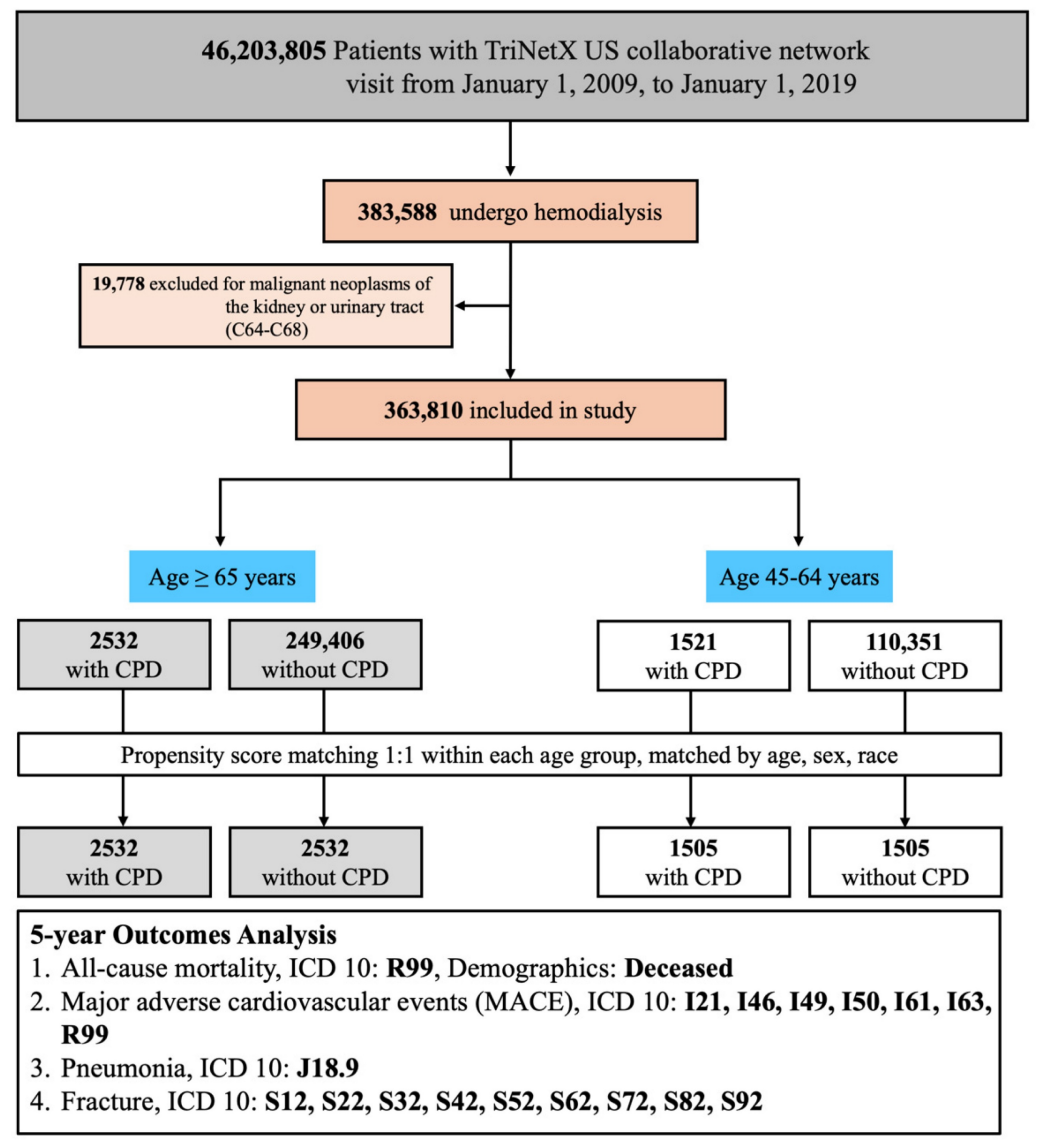
** Patient Selection Flowchart and Study Design.** Abbreviations: CPD, chronic periodontal disease; MACE, major adverse cardiovascular events; ICD-10, International Classification of Diseases, 10th Revision; PSM, propensity score matching.

This study included adult patients identified from the TriNetX US Collaborative Network, which comprises electronic health records from 143 healthcare organizations. Eligible patients had at least one clinical visit between January 1, 2009, and January 1, 2019. A total of 383,588 patients who underwent maintenance hemodialysis during this period were identified using procedural and diagnostic codes from Current Procedural Terminology (CPT: 1012752, 90937), the International Classification of Diseases, Ninth Revision, Clinical Modification (ICD-9-CM: 39.95), ICD-10-CM (Z99.2), and the Systematized Nomenclature of Medicine Clinical Terms (SNOMED-CT: 302497006). To improve cohort homogeneity, we excluded 19,778 patients with malignant neoplasms of the kidney or urinary tract (ICD-10-CM: C64-C68), resulting in a final cohort of 363,810 patients for further analysis. Patients were then stratified into two non-overlapping age groups based on their age at the index date: 45-64 years and ≥65 years. Within each age stratum, two mutually exclusive groups were defined based on the presence or absence of CPD. CPD was identified using ICD-10-CM codes K05.3, K05.30, K05.31, K05.32, K05.4, K05.5, and K05.6, diagnosed within 6 months before or up to 5 years after hemodialysis initiation. The comparison group included patients with no documented CPD diagnosis within the specified time window. To minimize confounding, 1:1 propensity score matching (PSM) was subsequently performed within each age group based on age at index (continuous), sex, and race/ethnicity (White, Asian, Black/African American). The final matched cohorts consisted of 2532 pairs in the ≥65-year group and 1505 pairs in the 45-64-year group (Figure 1).

### Index Date and Follow-Up Period

The index date was defined as the earliest date on which a patient met all eligibility criteria, including documented hemodialysis initiation and age group classification. CPD exposure status was subsequently determined based on diagnostic codes within a pre-specified time window relative to this index date. Each patient's observation window began the day after the index date and extended up to 1,825 days (5 years), or until death or loss to follow-up. Outcomes occurring before the index date were excluded from outcome analysis.

### Outcome Measures

The outcomes assessed were all-cause mortality, MACE, pneumonia, and fractures. All-cause mortality was identified using either a documented deceased status in demographic records or the ICD-10-CM code R99 (ill-defined and unknown cause of mortality). MACE was defined as a composite of acute myocardial infarction (I21), cardiac arrest (I46), cardiac arrhythmias (I49), heart failure (I50), nontraumatic intracerebral hemorrhage (I61), cerebral infarction (I63), or ill-defined mortality (R99). Pneumonia was identified by ICD-10-CM code J18 (pneumonia due to an unspecified organism). Fractures were defined using ICD-10-CM codes for traumatic or osteoporotic fractures involving cervical spine (S12), thoracic cage (S22), lumbar spine and pelvis (S32), upper extremities (S42, S52, S62), and lower extremities (S72, S82, S92).

### Propensity Score Matching

To reduce confounding bias, 1:1 PSM was conducted separately within each predefined age group (45-64 years and ≥65 years) using the greedy nearest-neighbor algorithm with a caliper width of 0.1 pooled standard deviations. Within each age stratum, the propensity score model included patient demographics, comorbid conditions, medication use, and key laboratory parameters. Although patients were stratified by age group, age at index was still included as a continuous covariate to adjust for residual within-stratum variation and to optimize covariate balance. Propensity scores were estimated using logistic regression. Covariate balance was assessed using standardized mean differences (SMDs), with values <0.1 indicating adequate balance. Baseline characteristics before and after matching are presented in Table 1 (age ≥65 years) and Table 2 (age 45-64 years).

### Statistical Analyses

Following matching, we assessed outcome differences between patients with and without CPD using two primary analytic approaches. First, we calculated absolute risks (event proportions), risk differences, risk ratios (RRs), and odds ratios (ORs) for each clinical outcome over the 5-year follow-up period. Patients with a documented history of a specific outcome prior to the index date were excluded from risk analyses for that outcome. Second, time-to-event data were analyzed using Kaplan-Meier survival curves to estimate cumulative incidence and compare survival probabilities between cohorts.

In TriNetX datasets, patients are censored at the date of their last recorded clinical activity within the observation window, which may lead to early exclusion from survival analyses. In contrast, fixed-time risk estimates generated by the TriNetX risk module retain these patients. To reconcile these methodological differences, we manually divided the 5-year follow-up into 90-day intervals (21 intervals totaling 1,890 days), calculated interval-specific risks (excluding duplicate events), and used cumulative event counts to generate Kaplan-Meier curves using the 'survival' package in R (version 4.4.2, Vienna, Austria). Survival curves were compared using the log-rank test.

For subgroup analyses, we estimated adjusted hazard ratios (aHRs) with 95% confidence intervals (CIs) using Cox proportional hazards regression models stratified by baseline characteristics (e.g., sex, diabetes, hypertension). Interaction terms were not included; therefore, subgroup-specific aHRs are presented descriptively to evaluate consistency across strata.

### Sensitivity Analyses

To assess the robustness of our findings, we conducted three sets of sensitivity analyses within the matched cohorts (see Supplementary Tables S1-S3). First, we re-estimated hazard ratios for each outcome using Cox models limited to patients with CPD diagnoses made within one year prior to hemodialysis initiation (Table S1). Second, we adjusted the Cox models for additional comorbidities—specifically hypertension, diabetes mellitus, and cerebrovascular disease—to determine whether CPD remained significantly associated with adverse outcomes after accounting for major chronic conditions (Table S2). Third, we further adjusted for inflammatory and nutritional biomarkers, including albumin, C-reactive protein (CRP), calcidiol, and urate, to evaluate whether observed associations were robust to biological confounding (Table S3).

## Results

### All-Cause Mortality

**Figure 2:**
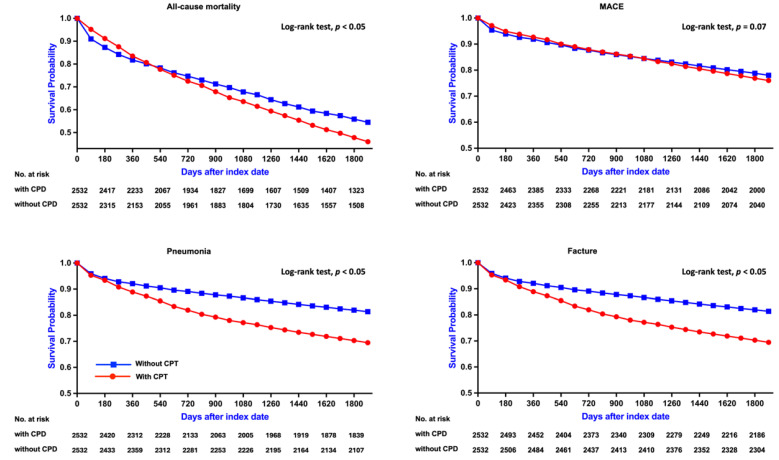
** Kaplan-Meier Survival Curves for Clinical Outcomes in Patients Aged ≥65 Years.** Survival curves comparing clinical outcomes between patients with CPD and without CPD among those aged ≥65 years. Statistically significant differences were observed for all-cause mortality, pneumonia, and fracture (log-rank p < 0.05). The difference in MACE-free survival did not reach statistical significance (log-rank p = 0.07). CPD, chronic periodontal disease; MACE, major adverse cardiovascular events.

Among hemodialysis patients aged ≥65 years, the presence of CPD was significantly associated with increased all-cause mortality over a 5-year follow-up period. After PSM, patients with CPD exhibited a higher risk of mortality compared to those without CPD (RR: 1.006, 95% CI: 0.919-1.102; OR: 1.210, 95% CI: 1.078-1.357; aHR: 1.126, 95% CI: 1.048-1.209). Kaplan-Meier survival analysis confirmed significantly lower survival probabilities in the CPD group (log-rank *p* < 0.05; Figure 2A). Subgroup analysis (Figure 4, left panel) did not reveal statistically significant differences across demographic or clinical strata.

**Figure 3:**
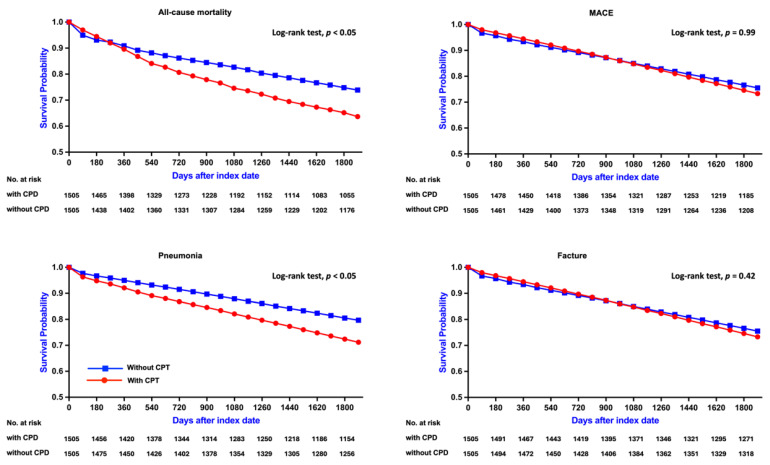
** Kaplan-Meier Survival Curves for Clinical Outcomes in Patients Aged 45-64 Years.** Survival curves comparing clinical outcomes between patients with CPD and without CPD among those aged 45-64 years. Significant differences were found for all-cause mortality and pneumonia (log-rank p < 0.05), while no significant differences were observed for MACE and fracture (p = 0.99 and p = 0.42, respectively). CPD, chronic periodontal disease; MACE, major adverse cardiovascular events.

**Figure 4:**
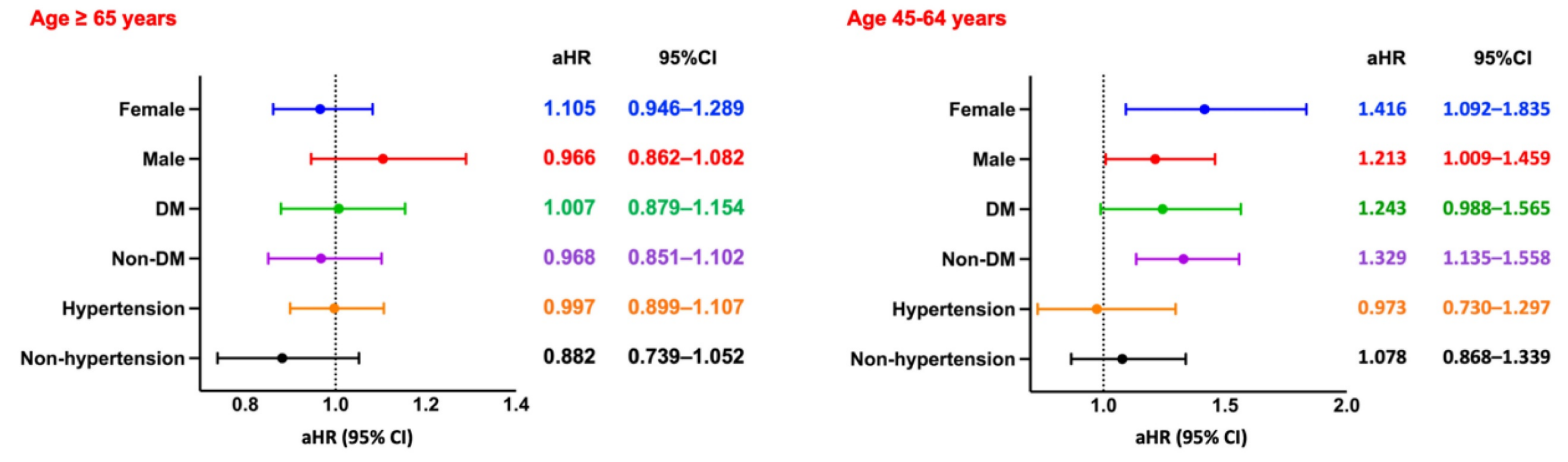
** Subgroup Analysis of All-Cause Mortality.** Forest plots of aHRs and 95% CIs for all-cause mortality across key subgroups by sex, diabetes status, and hypertension status, stratified by age group. DM, diabetes mellitus.

In contrast, among patients aged 45-64 years, CPD was associated with a more pronounced and statistically significant increase in all-cause mortality (RR: 1.477, 95% CI: 1.294-1.687; OR: 1.664, 95% CI: 1.401-1.976; aHR: 1.313, 95% CI: 1.129-1.527). Kaplan-Meier curves similarly demonstrated lower survival probabilities in the CPD group (log-rank *p* < 0.05; Figure 3A). Subgroup analysis (Figure 4, right panel) showed significantly elevated mortality risks among both sexes and among non-diabetic patients.

### Major Adverse Cardiovascular Events

**Figure 5:**
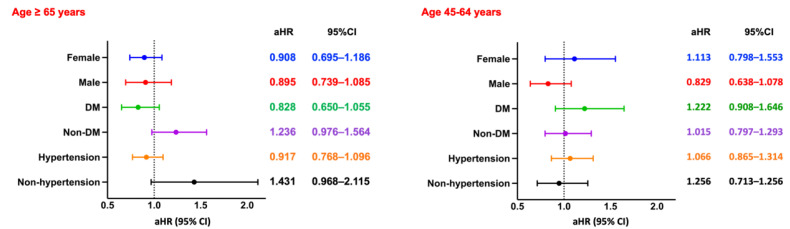
** Subgroup Analysis of MACE.** Forest plots of aHRs and 95% CIs for MACE across key subgroups by sex, diabetes status, and hypertension status, stratified by age group. DM, diabetes mellitus.

Among hemodialysis patients aged ≥65 years, the risk of MACE was slightly higher in those with CPD compared to those without, although the association did not reach statistical significance (RR: 1.111, 95% CI: 0.965-1.279; OR: 1.140, 95% CI: 0.957-1.359; aHR: 0.961, 95% CI: 0.821-1.124). Kaplan-Meier analysis similarly demonstrated no significant difference in cumulative MACE-free survival between the two groups (log-rank *p* = 0.07; Figure 2B). Subgroup analysis (Figure 5, left panel) did not identify any statistically significant differences; however, numerically elevated risks were observed among non-diabetic and non-hypertensive patients.

Among patients aged 45-64 years, the risk of MACE was likewise not significantly different between those with and without CPD (RR: 1.168, 95% CI: 0.964-1.416; OR: 1.204, 95% CI: 0.957-1.515; aHR: 1.005, 95% CI: 0.814-1.240). Kaplan-Meier curves confirmed the absence of a significant difference in MACE-free survival (log-rank *p* = 0.99; Figure 3B). Subgroup analysis (Figure 5, right panel) showed no statistically significant associations across any subgroup, as all confidence intervals included the null value.

### Pneumonia

**Figure 6:**
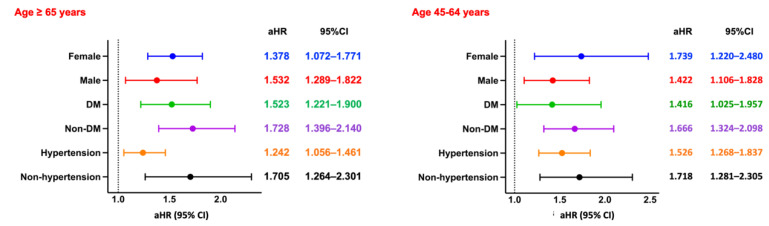
**Subgroup Analysis of Pneumonia.** Forest plots of aHRs and 95% CIs for pneumonia across key subgroups by sex, diabetes status, and hypertension status, stratified by age group. DM, diabetes mellitus.

Among hemodialysis patients aged ≥65 years, the incidence of pneumonia was significantly higher in the CPD group compared to those without CPD (RR: 1.521, 95% CI: 1.344-1.720; OR: 1.699, 95% CI: 1.455-1.985; aHR: 1.335, 95% CI: 1.163-1.533). Kaplan-Meier analysis demonstrated significantly lower pneumonia-free survival in the CPD group (log-rank *p* < 0.05; Figure 2C). Subgroup analysis (Figure 6, left panel) showed consistently elevated risks across both sexes, as well as among diabetic and non-diabetic patients, and among both hypertensive and non-hypertensive individuals.

A similar pattern was observed in patients aged 45-64 years, with the CPD group exhibiting a significantly higher pneumonia risk (RR: 2.021, 95% CI: 1.653-2.471; OR: 2.271, 95% CI: 1.799-2.867; aHR: 1.817, 95% CI: 1.463-2.256) and lower pneumonia-free survival (log-rank *p* < 0.05; Figure 3C). Subgroup analysis (Figure 6, right panel) revealed uniformly elevated risks across all strata, with particularly strong associations observed in females and in non-hypertensive patients.

### Fracture

**Figure 7:**
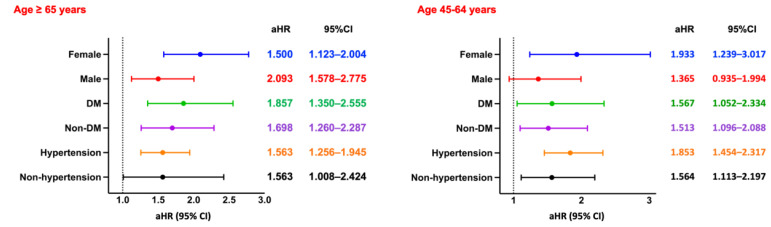
** Subgroup Analysis of Fracture.** Forest plots of aHRs and 95% CIs for fracture across key subgroups by sex, diabetes status, and hypertension status, stratified by age group. DM, diabetes mellitus.

Among hemodialysis patients aged ≥65 years, the incidence of fractures was significantly higher in the CPD group compared to the non-CPD group (RR: 1.868, 95% CI: 1.550-2.251; OR: 2.003, 95% CI: 1.629-2.462; aHR: 1.673, 95% CI: 1.375-2.036). Kaplan-Meier survival analysis revealed significantly lower fracture-free survival in the CPD group (log-rank *p* < 0.05; Figure 2D). Subgroup analysis (Figure 7, left panel) showed consistently elevated fracture risks across sex, diabetes status, and hypertension status.

A similar trend was observed in patients aged 45-64 years, with the CPD group exhibiting a significantly higher fracture risk (RR: 1.722, 95% CI: 1.304-2.275; OR: 1.795, 95% CI: 1.332-2.420). However, Kaplan-Meier analysis indicated no statistically significant difference in fracture-free survival between the two groups (log-rank *p* = 0.42; Figure 3D). Subgroup analysis (Figure 7, right panel) revealed significantly increased fracture risks among females, diabetic and non-diabetic patients, and individuals with and without hypertension, whereas no significant association was observed in males.

### Sensitivity Analyses

In the matched cohorts, CPD was significantly associated with increased risks of all-cause mortality, pneumonia, and fractures—but not MACE—across both age groups (Table S1). In the second sensitivity analysis, aHRs were further controlled for additional comorbidities, including hypertension, cerebrovascular disease, and diabetes mellitus. CPD remained significantly associated with pneumonia and fracture risks and was also linked to higher all-cause mortality in patients aged 45-64 years (Table S2). In the third analysis, aHRs were additionally adjusted for inflammatory and nutritional biomarkers, including albumin, CRP, calcidiol, and urate. Under this model, the associations with pneumonia persisted in both age groups, while the associations with all-cause mortality and fracture remained statistically significant only in the 45-64-year group (Table S3).

## Discussion

This study provides an age-stratified analysis exploring the associations between CPD and major clinical outcomes among patients undergoing maintenance hemodialysis. CPD was significantly associated with increased risks of all-cause mortality and pneumonia across both age groups, with more pronounced effects observed in younger patients for these outcomes. A significant association with fracture risk was observed only among older patients, while no significant associations were identified between CPD and MACE in either age group. These findings emphasize the systemic impact of CPD and the importance of incorporating age-specific considerations into risk assessment and clinical management strategies for hemodialysis patients.

For all-cause mortality, CPD was significantly associated with risk in both older (≥65 years) and younger (45-64 years) cohorts, with a stronger effect in the younger group. Kaplan-Meier analyses consistently showed reduced survival in patients with CPD. Subgroup analyses in the elderly revealed no significant effect modifications, whereas in the younger group risks were elevated across sexes and in non-diabetic patients, suggesting a more prominent impact in middle-aged dialysis populations. These results align with prior studies. A Danish nationwide cohort (mean age 57.3) reported an adjusted incidence rate ratio of 2.70 (95% CI: 2.60-2.81) for mortality in patients with periodontitis versus controls 16. Similarly, a prospective hemodialysis study (mean age 58.8) found higher mortality with moderate and severe periodontitis (HRs: 1.39 and 1.83, respectively) 11. Although the association persisted in the elderly, the smaller effect size may reflect competing comorbidities influencing mortality.

This study found no significant association between CPD and MACE in either older (≥65 years) or younger (45-64 years) cohorts. Kaplan-Meier analysis and risk estimates were similarly non-significant, and subgroup analyses also showed no significant associations, though numerically higher risks appeared in non-diabetic and non-hypertensive patients. These findings underscore the complexity of cardiovascular risk in this population. Mechanistically, the lack of association may reflect the high baseline inflammatory burden of hemodialysis, marked by elevated interleukin-6 (IL-6), tumor necrosis factor-alpha (TNF-α), and CRP, which may mask the contribution of CPD to atherogenesis and plaque destabilization 17. In elderly patients, this baseline inflammatory burden, combined with other comorbidities, further reduces the detectability of any additional cardiovascular risk conferred by CPD.

Although our findings were null, prior studies on CPD and cardiovascular outcomes show mixed results. A systematic review reported relative risks of 1.14-2.20 for cardiovascular disease with moderate to severe periodontitis 18, contrasting with our results and likely reflecting lower baseline inflammation in the general population. An international consensus also noted that CPD may promote cardiovascular disease via chronic inflammation, bacteremia, and cytokine-mediated endothelial dysfunction 19, mechanisms that may be obscured in dialysis patients. In dialysis-specific cohorts, results remain inconsistent: a U.S. study of 168 patients reported markedly higher cardiovascular mortality with periodontitis (HR: 5.00; 95% CI: 1.20-19.10) 15 , while a larger U.S. study of 912 hemodialysis patients found a modest but significant association (HR: 1.41; 95% CI: 1.05-1.90) 12. In contrast, the multinational ORAL-D study found no significant association (HR: 1.06; 95% CI: 0.87-1.29) 11, consistent with our findings and suggesting that CPD's cardiovascular impact in ESRD may be limited.

Variation across studies likely reflects differences in methodology, populations, and exposure classification. Our use of ICD-10 coding may underreport CPD severity, attenuating effects, whereas studies showing significant results often relied on detailed clinical assessments. Nonetheless, our study incorporated rigorous PSM and adjustments for albumin, CRP, vitamin D, and urate, which were not uniformly included in prior work. The relatively short follow-up (~2.5 years) may also have limited long-term detection. Overall, despite divergence from some smaller or general-population studies, our findings align with larger dialysis-specific cohorts, suggesting that under high inflammatory burden, CPD's cardiovascular contribution is modest or masked by competing risks.

In contrast to MACE, CPD was significantly associated with pneumonia risk across both age groups, underscoring its systemic inflammatory impact and role in pulmonary complications—a major cause of morbidity and mortality in hemodialysis. Pneumonia was selected as a primary outcome due to strong clinical relevance and evidence linking periodontal disease to respiratory infections through aspiration of oral pathogens and systemic inflammation. A 7-year cohort study of hemodialysis patients reported pneumonia-related mortality of 18.5% with periodontal disease versus 3.4% without (adjusted HR 3.49; 95% CI: 1.14-10.64) 13, while a meta-analysis showed a 44% increased risk of nosocomial pneumonia in periodontitis 20. Subgroup analyses revealed consistent associations across demographic and clinical strata, with significant risks observed in both older (≥65 years) and younger (45-64 years) groups. Mechanistically, periodontal pathogens can enter the bloodstream, impair host defenses, and modulate respiratory mucosa via IL-6, TNF-α, and CRP 21-24. Elderly dialysis patients are particularly vulnerable due to reduced pulmonary defenses, while younger patients may mount exaggerated inflammatory responses, amplifying pneumonia risk. These findings emphasize the pervasive pulmonary impact of CPD and highlight the need for preventive oral health interventions in hemodialysis populations.

Taken together, these findings also raise the question of why CPD was associated with more pronounced risks of mortality and pneumonia in younger rather than older patients. Several mechanisms may explain this observation. Older hemodialysis patients experience immunosenescence, characterized by diminished T-cell function, impaired natural killer cell activity, and attenuated innate immune responses 25, 26. While this immunologic decline increases baseline infection susceptibility, it may also blunt the incremental systemic inflammatory response induced by CPD, thereby attenuating its detectable impact. By contrast, younger patients are more likely to mount robust but dysregulated inflammatory reactions to periodontal pathogens, including exaggerated production of IL-6 and TNF-α, which amplify systemic injury. In addition, elderly patients face multiple competing risks—such as cardiovascular disease, malignancy, malnutrition, and frailty—that dominate clinical outcomes and may overshadow the contribution of CPD 27, 28. Finally, while chronic inflammation is pervasive across all dialysis populations 29, 30, the incremental effect of CPD may be more evident in younger patients who have fewer comorbidities and lower baseline inflammatory activity. These considerations provide a biologically plausible explanation for the stronger associations of CPD with mortality and pneumonia observed in the younger cohort.

The observed association between CPD and fractures highlights the systemic impact of periodontal inflammation on bone health in hemodialysis patients. This endpoint was included due to its high incidence and clinical relevance, supported by biological plausibility. Mechanistically, CPD elevates pro-inflammatory cytokines that activate the receptor activator of nuclear factor kappa-B ligand /osteoprotegerin pathway, enhance osteoclastogenesis, and suppress osteoblast function, thereby promoting generalized bone loss 7, 31. Periodontal pathogens and their byproducts may also trigger systemic immune responses and expand osteoclast precursors, linking oral infection to skeletal fragility 31. CPD and osteoporosis further share risk factors such as aging, vitamin D deficiency, smoking, and hormonal changes, which synergistically increase fracture susceptibility 32. Epidemiologic studies support these mechanisms, showing higher odds of osteoporosis and fractures in patients with periodontitis (OR 1.5-2.2) 33-35, with vertebral fractures reported in over 40% of elderly CPD patients versus 25% without CPD 36.

Direct data linking CPD severity to osteoporosis or renal osteodystrophy in hemodialysis patients are limited, but indirect evidence is strong. In the general population, severe periodontitis correlates with lower bone mineral density and higher fracture risk, while in dialysis cohorts, longer dialysis duration is a recognized risk factor for renal osteodystrophy and bone loss through chronic kidney disease-mineral and bone disorder (CKD-MBD), malnutrition, and inflammation 37-39. Thus, the convergence of CPD-driven systemic inflammation with CKD-related skeletal fragility provides a compelling biologic rationale for our findings. In our analyses, CPD was significantly associated with fracture risk in older adults (≥65 years), with consistent effects across sex and comorbidities. In the 45-64 group, elevated risks were also observed, particularly among females, though survival differences were less pronounced, likely reflecting higher baseline bone density and shorter dialysis vintage 40, 41. These results align with prior evidence linking osteoporosis and CPD, especially among postmenopausal women 7, 31, 42​.

Although effect sizes were moderate, they remain clinically meaningful given the high baseline risks and limited reserves in hemodialysis patients. For instance, an aHR of 1.313 for mortality in the 45-64-year group and a pneumonia risk >2.0 translate into substantial absolute event burden. Similarly, fracture risk (RR up to 1.868 in ≥65-year group) reflects significant skeletal vulnerability. These results align with studies linking CPD to systemic inflammation, immune dysfunction, and adverse outcomes in dialysis cohorts 43-45, showing that even modest risks can carry important clinical implications.

Clinically, our findings support integrating periodontal care into dialysis management. CPD is a persistent, modifiable inflammatory source that worsens immune regulation, nutrition, and pathogen translocation 46. Interventional studies show periodontal therapy reduces CRP and improves serum albumin in hemodialysis patients 47. The strong association with pneumonia supports dental evaluation in infection prevention 48, while fracture links suggest incorporating oral health into bone risk assessment 49, 50. Given the heightened vulnerabilities observed among elderly, female, and diabetic subpopulations, tailored interventions are warranted, and multidisciplinary collaboration among nephrology, dental, and geriatric specialists is essential for optimizing outcomes.

One key limitation is the identification of CPD using ICD-10-CM codes within a defined window around dialysis initiation. While this enables large-scale analysis, the accuracy of ICD codes for periodontal disease is variable, with sensitivity and specificity differing across conditions and settings 51. Codes lack detail on staging or severity and may underreport CPD if dental care occurs outside TriNetX. Nevertheless, prior studies show ICD-coded CPD correlates with systemic diseases such as hypertension and diabetes 52. In psychiatric research, concordance with chart-confirmed diagnoses is ~50%, underscoring the need for validation 53. To reduce misclassification, we used a fixed diagnostic window and sensitivity analyses restricted to cases within one year before dialysis. The consistency of results across matched cohorts, supported by biological plausibility, lends credibility to our approach. Other limitations include residual confounding since PSM initially matched only age, sex, and race, without socioeconomic or oral health variables. Structured EHR reliance, absence of CPD treatment history, and limited biomarker data may also bias results. Strict inclusion criteria and age stratification reduced sample size, while CPD underreporting in U.S. EHRs may persist. We excluded only renal and urinary tract cancers to avoid severe comorbidity bias. Initial matching was limited to basic demographics, though later sensitivity analyses added comorbidities and biomarkers. Finally, the predominance of U.S.-based TriNetX cases may limit generalizability and reflect healthcare access disparities.

## Conclusion

This study demonstrates that CPD is significantly associated with increased risks of mortality, pneumonia, and fractures among hemodialysis patients, with notable age- and subgroup-specific variations. In patients aged 45-64 years, CPD was more strongly associated with all-cause mortality and pneumonia, particularly among both sexes and non-diabetic individuals. In contrast, in patients aged ≥65 years, CPD showed a pronounced association with fracture risk. No significant association was observed between CPD and MACE in either age group. These findings highlight the systemic impact of CPD in the dialysis population and underscore the importance of integrating routine periodontal assessment and care into the multidisciplinary management of hemodialysis patients, especially in vulnerable subgroups.

## Supplementary Material

Supplementary figures and tables.

## Figures and Tables

**Table 1:** Baseline characteristics of hemodialysis patients aged ≥65 with or without chronic periodontal disease, before and after propensity score matching.

Participants^a^	Before Matching				After Matching			
Characteristic	With CPD (n=2532)	Without CPD (n=249,406)	*p* value	SMD	With CPD (n=2532)	Without CPD (n=2532)	*p* value	SMD
Demographics								
Age at index, years (mean ± SD)	67.0 ± 9.4	68.4 ± 9.3	<0.01	0.14	67.1 ± 9.4	66.9 ± 9.3	0.49	0.02
White (%)	12.6%	36.1%	<0.01	0.57	13.3%	11.0%	0.032	0.07
Female (%)	35.7%	42.5%	<0.01	0.14	35.9%	35.2%	0.62	0.02
Black or African American (%)	13.4%	17.8%	<0.01	0.12	13.9%	13.8%	0.93	0.01
Asian (%)	68.4%	27.2%	<0.01	0.91	66.9%	70.6%	0.01	0.08
Diagnoses (%)								
Hypertensive diseases	51.6%	36.4%	<0.01	0.31	51.6%	52.7%	0.46	0.02
Cerebrovascular diseases	13.7%	7.4%	<0.01	0.21	13.4%	13.2%	0.86	0.01
Acute kidney failure and CKD	45.5%	40.9%	<0.01	0.09	45.6%	43.8%	0.22	0.04
Diabetes mellitus	34.3%	24.9%	<0.01	0.21	34.7%	33.4%	0.37	0.03
Gingivitis and periodontal diseases	21.1%	0.1%	<0.01	0.73	13.3%	11.2%	0.02	0.07
Dental caries	13.3%	0.2%	<0.01	0.54	9.9%	9.3%	0.45	0.02
Diseases of pulp/periapical tissues	6.0%	0.1%	<0.01	0.35	4.2%	3.8%	0.50	0.02
Ischemic heart diseases	23.4%	16.6%	<0.01	0.17	23.4%	23.0%	0.75	0.01
Diseases of arteries/arterioles	12.9%	9.8%	<0.01	0.09	12.7%	12.4%	0.756	0.01
Malnutrition	2.8%	2.9%	0.69	0.01	2.8%	2.4%	0.31	0.03
Diseases of oral cavity/salivary glands	40.4%	2.9%	<0.01	1.02	34.0%	33.3%	0.60	0.02
Diseases of esophagus/stomach/duodenum	28.8%	11.8%	<0.01	0.43	28.1%	29.7%	0.23	0.04
Medications (%)								
Beta blockers	28.6%	23.5%	<0.01	0.12	28.9%	28.6%	0.82	0.01
CCB	32.6%	19.0%	<0.01	0.32	32.2%	32.5%	0.83	0.01
ARB	18.1%	7.4%	<0.01	0.33	18.1%	17.8%	0.82	0.01
Alpha blockers	15.8%	6.7%	<0.01	0.29	14.9%	15.5%	0.57	0.02
ACE inhibitors	8.4%	7.5%	0.12	0.03	8.5%	7.8%	0.36	0.03
Antianginals	14.2%	10.2%	<0.01	0.12	13.9%	13.6%	0.76	0.01
Analgesics	67.5%	38.1%	<0.01	0.63	65.6%	67.0%	0.32	0.03
Sedative/hypnotics	37.2%	20.8%	<0.01	0.37	36.7%	37.7%	0.50	0.02
Antidepressants	12.4%	8.5%	<0.01	0.13	12.2%	12.4%	0.86	0.01
Antacids	33.8%	23.9%	<0.01	0.22	33.3%	33.1%	0.90	0.01
Antiulcer agents	20.3%	7.7%	<0.01	0.37	19.7%	20.8%	0.36	0.03
Antilipidemic agents	24.8%	18.4%	<0.01	0.15	24.7%	23.8%	0.47	0.02
Diuretics	30.1%	22.7%	<0.01	0.17	29.6%	28.9%	0.58	0.02
Laboratory values (mean ± SD)								
Albumin (g/dL)	3.7 ± 0.7	3.3 ± 0.8	<0.01	0.60	3.7 ± 0.7	3.4 ± 0.8	<0.01	0.39
HbA1c (%)	6.6 ± 1.4	6.7 ± 1.7	0.06	0.08	6.6 ± 1.5	6.7 ± 1.6	0.15	0.08
Calcidiol (ng/mL)	25.4 ± 14.7	25.5 ± 15.5	0.99	<0.01	26.1 ± 14.9	25.6 ± 14.3	0.86	0.04
C-reactive protein (mg/L)	13.3 ± 32.7	41.5 ± 72.0	<0.01	0.50	13.5 ± 33.0	15.9 ± 44.4	0.32	0.06
ESR (mm/h)	56.7 ± 32.9	56.3 ± 35.3	0.93	0.01	57.4 ± 33.3	54.4 ± 33.4	0.62	0.09
Urate (mg/dL)	6.4 ± 2.0	7.1 ± 2.9	<0.01	0.30	6.4 ± 2.0	6.6 ± 2.6	0.15	0.10
BMI (kg/m²)	25.8 ± 5.9	28.4 ± 7.3	<0.01	0.39	25.8 ± 5.9	26.1 ± 6.3	0.36	0.04
SBP (mmHg)	128.8 ± 26.1	127.4 ± 28.9	0.24	0.05	128.5 ± 26.0	129.5 ± 26.6	0.52	0.04
DBP (mmHg)	68.3 ± 13.4	65.2 ± 15.8	<0.01	0.21	68.4 ± 13.5	66.7 ± 14.6	0.04	0.11

Abbreviations: ACEI, angiotensin-converting enzyme inhibitor; ARB, angiotensin II receptor blocker; BMI, body mass index; CCB, calcium channel blocker; CKD, chronic kidney disease; CPD, Chronic periodontal disease; CRP, C-reactive protein; DBP, diastolic blood pressure; ESR, Erythrocyte sedimentation rate; HbA1c, hemoglobin A1c; SBP, systolic blood pressure; SMD, standardized mean difference. a. Data are presented as % unless otherwise indicated.

**Table 2:** Baseline characteristics of hemodialysis patients aged 45-64 with or without chronic periodontal disease, before and after propensity score matching.

Participants^a^	Before Matching				After Matching			
Characteristic	With CPD (n=1521)	Without CPD (n=110,351)	*p* value	SMD	With CPD (n=1505)	Without CPD (n=1505)	*p* value	SMD
Demographics								
Age at index, years (mean ± SD)	46.3 ± 5.6	46.4 ± 6.1	0.70	0.01	46.3 ± 5.6	46.5 ± 5.9	0.46	0.03
White (%)	14.9%	29.9%	<0.01	0.37	15.1%	13.8%	0.34	0.04
Female (%)	36.6%	41.7%	<0.01	0.10	36.6%	34.9%	0.35	0.04
Black or African American (%)	19.0%	23.9%	<0.01	0.12	19.3%	17.8%	0.31	0.04
Asian (%)	59.8%	30.2%	<0.01	0.62	59.3%	61.2%	0.30	0.04
Diagnoses (%)								
Hypertensive diseases	32.1%	27.7%	<0.01	0.10	31.7%	30.0%	0.33	0.04
Cerebrovascular diseases	4.1%	3.8%	0.54	0.02	4.0%	3.6%	0.56	0.02
Acute kidney failure and CKD	33.7%	37.1%	0.01	0.07	33.7%	32.5%	0.47	0.03
Diabetes mellitus	19.3%	17.4%	0.06	0.05	19.0%	17.3%	0.26	0.04
Gingivitis and periodontal diseases	15.8%	0.2%	<0.01	0.60	12.4%	12.1%	0.77	0.01
Dental caries	8.6%	0.4%	<0.01	0.41	6.2%	5.5%	0.42	0.03
Diseases of pulp/periapical tissues	2.9%	0.2%	<0.01	0.22	2.3%	2.2%	0.80	0.01
Ischemic heart diseases	9.2%	7.4%	0.01	0.07	8.9%	7.4%	0.13	0.06
Diseases of arteries/arterioles	6.5%	5.2%	0.02	0.06	6.5%	5.7%	0.34	0.04
Malnutrition	2.7%	2.5%	0.57	0.01	2.7%	1.7%	0.07	0.07
Diseases of oral cavity/salivary glands	25.2%	3.0%	<0.01	0.67	22.2%	21.9%	0.82	0.01
Diseases of esophagus/stomach/duodenum	13.1%	9.5%	<0.01	0.12	13.1%	12.9%	0.87	0.01
Medications (%)								
Beta blockers	21.1%	19.2%	0.05	0.05	21.0%	19.2%	0.24	0.04
CCB	18.6%	14.9%	<0.01	0.10	18.5%	18.3%	0.92	0.01
ARB	6.7%	4.8%	0.01	0.08	6.5%	6.0%	0.59	0.02
Alpha blockers	3.9%	2.7%	0.01	0.06	4.0%	3.7%	0.77	0.01
ACE inhibitors	8.0%	6.4%	0.01	0.06	7.9%	6.1%	0.06	0.07
Antianginals	5.8%	5.5%	0.56	0.01	5.9%	5.0%	0.28	0.04
Analgesics	48.9%	33.6%	<0.01	0.32	47.8%	48.0%	0.91	0.01
Sedative/hypnotics	26.5%	18.5%	<0.01	0.19	26.0%	23.4%	0.11	0.06
Antidepressants	8.2%	7.2%	0.17	0.04	7.9%	7.1%	0.39	0.03
Antacids	21.1%	18.2%	0.01	0.07	20.7%	18.1%	0.08	0.07
Antiulcer agents	10.5%	7.1%	<0.01	0.12	10.7%	10.0%	0.54	0.02
Antilipidemic agents	12.3%	11.2%	0.18	0.04	12.4%	11.0%	0.27	0.04
Diuretics	23.7%	17.0%	<0.01	0.17	22.9%	22.3%	0.72	0.01
Laboratory values (mean ± SD)								
Albumin (g/dL)	3.7 ± 0.8	3.4 ± 0.8	<0.01	0.43	3.7 ± 0.8	3.7 ± 0.8	0.67	0.02
HbA1c (%)	6.9 ± 2.1	6.9 ± 2.2	0.57	0.04	6.9 ± 2.1	6.8 ± 2.1	0.45	0.07
Calcidiol (ng/mL)	19.3 ± 12.2	20.2 ± 14.1	0.65	0.07	19.3 ± 12.1	21.2 ± 14.4	0.48	0.14
CRP (mg/L)	27.3 ± 62.7	37.6 ± 70.9	0.03	0.15	28.0 ± 63.9	17.5 ± 45.2	0.06	0.19
ESR (mm/h)	65.4 ± 37.5	58.2 ± 37.0	0.21	0.19	64.8 ± 37.7	73.5 ± 35.3	0.30	0.24
Urate (mg/dL)	6.4 ± 2.1	7.0 ± 2.9	0.01	0.25	6.4 ± 2.0	6.6 ± 2.7	0.35	0.10
BMI (kg/m²)	26.6 ± 7.0	28.9 ± 8.2	<0.01	0.30	26.5 ± 6.9	26.6 ± 7.4	0.91	0.01
SBP (mmHg)	134.3 ± 27.2	130.3 ± 29.3	0.01	0.14	134.2 ± 27.1	136.1 ± 27.5	0.39	0.07
DBP (mmHg)	76.4 ± 16.8	73.5 ± 17.7	0.01	0.17	76.4 ± 16.8	77.2 ± 16.5	0.57	0.05

Abbreviations: ACEI, angiotensin-converting enzyme inhibitor; ARB, angiotensin II receptor blocker; BMI, body mass index; CCB, calcium channel blocker; CKD, chronic kidney disease; CPD, Chronic periodontal disease; CRP, C-reactive protein; DBP, diastolic blood pressure; ESR, Erythrocyte sedimentation rate; HbA1c, hemoglobin A1c; SBP, systolic blood pressure; SMD, standardized mean difference. a. Data are presented as % unless otherwise indicated.

## Abbreviations

ACEI: angiotensin-converting enzyme inhibitor
aHR: adjusted hazard ratio
ARB: angiotensin II receptor blocker
BMI: body mass index
CCB: calcium channel blocker
CKD: chronic kidney disease
CPD: chronic periodontal disease
CRP: C-reactive protein
DBP: diastolic blood pressure
DM: diabetes mellitus
ESR: erythrocyte sedimentation rate
ESRD: end-stage renal disease
HbA1c: hemoglobin A1c
MACE: major adverse cardiovascular events
OR: odds ratio
PSM: propensity score matching
RR: risk ratio
SBP: systolic blood pressure
TNF-α: tumor necrosis factor-alpha
