# Supplementary material for: Age-stratified Associations between Chronic Periodontal Disease and Clinical Outcomes in Hemodialysis Patients: Mortality, Pneumonia, and Fractures
Source: Int J Med Sci. 2025 Oct 24;22(16):4469–82. doi: 10.7150/ijms.116133 (PMC12595327; doi:10.7150/ijms.116133)
Supplement: Supplementary file 1 — Supplementary figures and tables. [file ijmsv22p4469s1.pdf]

Table S1. Sensitivity analysis of clinical outcomes in hemodialysis patients with chronic periodontal disease diagnosed within one year prior to dialysis initiation.

**Age group**	**Outcomes**	**Patients with CPD (n/N)**	**Patients without CPD (n/N)**	**Adjusted hazard ratio (95% CI)[a]**	***p* value**
**≥65 years**	All-cause mortality	503/989	358/993	1.353 (1.181-1.549)	<0.001
	MACE	117/536	139/690	0.984 (0.769-1.258)	0.896
	Pneumonia	205/686	157/884	1.585 (1.287-1.951)	<0.001
	Fracture	106/849	50/929	2.199 (1.571-3.079)	<0.001
**45–64 years**	All-cause mortality	218/543	124/565	1.680 (1.347-2.094)	<0.001
	MACE	74/350	75/457	1.211 (0.878-1.670)	0.242
	Pneumonia	109/413	50/517	2.674 (1.913-3.738)	<0.001
	Fracture	50/519	24/544	2.042 (1.255-3.322)	0.003

CPD, Chronic periodontal disease; CI, Confidence interval; MACE, major adverse cardiovascular events.

a. Hazard ratios were adjusted for age at index, sex, race.

Table S2. Sensitivity analysis of clinical outcomes in hemodialysis patients with chronic periodontal disease, adjusted for additional comorbidities.

**Age group**	**Outcomes**	**Patients with CPD (n/N)**	**Patients without CPD (n/N)**	**Adjusted hazard ratio (95% CI)[a]**	***p* value**
**≥65 years**	All-cause mortality	969/2,407	920/2,410	0.930 (0.850–1.018)	0.117
	MACE	278/1,409	318/1,602	0.870 (0.740–1.022)	0.089
	Pneumonia	443/1,741	353/2,039	1.309 (1.138–1.505)	< 0.001
	Fracture	244/1,851	169/2,172	1.515 (1.245–1.844)	< 0.001
**45–64 years**	All-cause mortality	377/1,337	266/1,355	1.289 (1.102–1.508)	0.001
	MACE	147/915	180/1,040	0.797 (0.641–0.990)	0.040
	Pneumonia	194/1,020	123/1,206	1.718 (1.370–2.153)	< 0.001
	Fracture	91/1,224	53/1,289	1.632 (1.163–2.290)	0.004

CPD, Chronic periodontal disease; CI, Confidence interval; MACE, major adverse cardiovascular events.

a. Hazard ratios were adjusted for age at index, sex, race, hypertension, cerebrovascular disease, and diabetes mellitus.

Table S3. Sensitivity analysis of clinical outcomes in hemodialysis patients with chronic periodontal disease, adjusted for comorbidities and laboratory values.

**Age group**	**Outcomes**	**Patients with CPD (n/N)**	**Patients without CPD (n/N)**	**Adjusted hazard ratio (95% CI)[a]**	***p* value**
**≥65 years**	All-cause mortality	966/2,409	938/2,407	0.916 (0.837–1.002)	0.056
	MACE	278/1,410	293/1,607	0.945 (0.802–1.113)	0.497
	Pneumonia	442/1,743	320/2,007	1.425 (1.234–1.646)	<0.001
	Fracture	246/1,853	182/2,150	1.414 (1.167–1.713)	<0.001
**45–64 years**	All-cause mortality	394/1,394	306/1,410	1.164 (1.003–1.352)	0.046
	MACE	155/937	172/1,076	0.891 (0.717–1.107)	0.298
	Pneumonia	199/1,061	154/1,252	1.346 (1.091–1.662)	0.005
	Fracture	93/1,274	84/1,352	1.035 (0.770–1.390)	0.821

CPD, Chronic periodontal disease; CI, Confidence interval; MACE, major adverse cardiovascular events.

a. Hazard ratios were adjusted for age at index, sex, race, hypertension, cerebrovascular disease, diabetes mellitus, albumin, C- reactive protein, calcidiol, and urate.
